# Idiopathic Calcinosis Cutis in a Child: Report of a Rare Case

**DOI:** 10.7759/cureus.34254

**Published:** 2023-01-26

**Authors:** Muhannad Al Wadany, Feras Al Wadany, Abdulelah Almousa, Farah Almoussa, Ahlam Alharbi

**Affiliations:** 1 General Practice, King Faisal University, Hofuf, SAU; 2 General Practice, Alfaisal University, Riyadh, SAU; 3 Family Medicine, Primary Health Care, Riyadh, SAU

**Keywords:** idiopathic, case report, knee lesion, calcification, calcinosis cutis

## Abstract

Calcinosis cutis is characterized by the deposition of calcium salts in the skin and subcutaneous tissue. Calcinosis cutis has different types, but the idiopathic type is considered the rarest type. We present the case of a 10-year-old boy who presented with a skin lesion on his right knee. No other similar nodules were noted elsewhere in the body. The lesion was first noted one year ago, and it slightly increased in size. The lesion was not pruritic and did not ulcerate. No history of previous trauma was provided. On physical examination, a nontender, firm, immobile, reddish, solitary nodule of 2 cm in diameter was observed on the extensor surface of the right knee. The patient underwent complete laboratory investigations that included hematological, biochemical, and immunological parameters, which yielded normal results. Excisional biopsy was performed, and histopathological examination revealed well-circumscribed deposits of basophilic materials in the subcutaneous tissue that is consistent with calcium deposits of calcinosis cutis. Idiopathic calcinosis cutis is a rare condition in children, particularly if it has a unilateral distribution. Proper evaluation should be performed to rule out any associated metabolic or systemic disorders that may alter the management pathway.

## Introduction

Calcinosis cutis is a dermatological condition that is characterized by abnormal deposition of calcium salts in the skin and subcutaneous tissue [1]. This condition has five unique types: idiopathic, dystrophic, metastatic, iatrogenic, and calciphylaxis. In the idiopathic type, the calcification occurs with normal electrolyte levels with no associated tissue damage [1]. The dystrophic type is the most common and has normal electrolyte levels but is associated with underlying diseases, such as dermatomyositis, systemic sclerosis, systemic lupus erythematosus, or mixed connective tissue disease. In the metastatic type, there are abnormal calcium and phosphorus levels. The iatrogenic type occurs through the administration of medications that contain calcium or phosphorus, resulting in abnormal precipitation of calcium salts. The calciphylaxis type is a unique type that occurs in chronic renal failure and involves calcification of the small- and medium-sized arteries [1,2]. Identification of the type of calcinosis cutis is crucial for accurate management [1,2]. The management lines include smoking cessation along with several medications, including diltiazem, bisphosphonates, warfarin, aluminum hydroxide, ceftriaxone, and other treatments [1,2]. Limited cases of idiopathic calcinosis have been reported. Furthermore, idiopathic calcinosis cutis is very rare to have unilateral distribution [2,3]. Here, we report the case of a young boy with idiopathic calcinosis cutis involving the knee.

## Case presentation

We present the case of a 10-year-old boy who came to our outpatient clinic with a complaint of a skin lesion on his right knee. The lesion was first noted one year ago, and it slightly increased in size. The lesion was not pruritic and did not ulcerate. No history of previous trauma was provided. No other similar lesions were noted. The lesion was not associated with swelling, pain, or restricted range of motion of the knee. The patient’s medical history was significant for a sickle cell trait. Otherwise, the past surgical and social history were insignificant.

**Figure 1:**
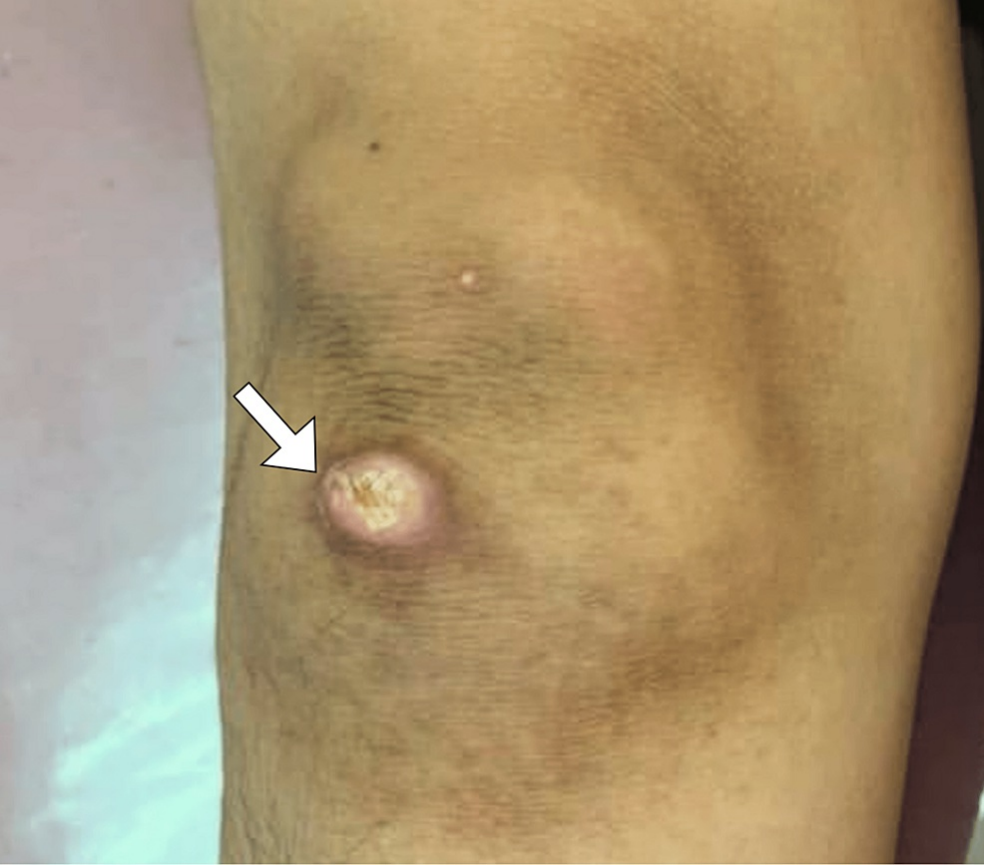
A gross photograph shows a nodule (arrow) over the extensor surface of the right knee.

On physical examination, a nontender, firm, immobile, reddish, solitary nodule of 2 cm in diameter was observed on the extensor surface of the right knee (Figure 1). No other skin lesions were identified. The knee examination was normal. The nodule was not associated with muscle weakness to suggest dermatomyositis.

The patient had normal results of basic laboratory investigations, including complete blood count, hepatic profile, urea and electrolytes, C-reactive protein, and erythrocyte sedimentation rate. In particular, the calcium level was 9.8 mg/dL. The hormonal profile that included thyroid-stimulating hormone (3.1 mIU/L), parathyroid hormone (20 pg/mL), calcitonin (7.1 pg/mL), and vitamin D (25 ng/mL), all yielded normal results. Furthermore, immunological investigations that included C3, C4, antinuclear antibody (ANA), antineutrophil cytoplasmic autoantibodies (ANCA), anti-dsDNA, anti-Sm, anti-Ro, and anti-La antibodies, were all negative.

**Figure 2:**
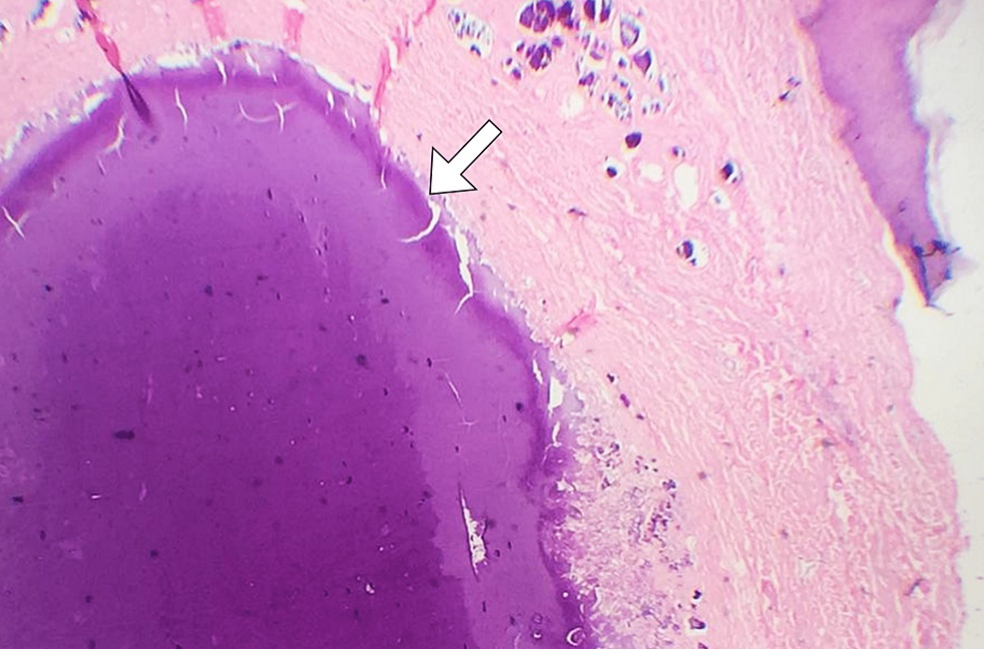
A high-power field histopathological image shows the lesion with an area of basophilic calcified material (arrow) in the skin.

A biopsy of the nodule was performed. Histopathological examination revealed well-circumscribed deposits of basophilic materials in the subcutaneous tissue that is consistent with calcium deposits of calcinosis cutis (Figure 2). The patient had a regular follow-up in the rheumatology clinic for two years, and he had no new lesions identified.

## Discussion

We report an unusual case of unilateral idiopathic calcinosis cutis in a child. The idiopathic form of calcinosis cutis is the rarest type of calcinosis cutis. By definition, it has normal calcium and phosphorus levels, with no associated systemic or metabolic disorders. Idiopathic calcinosis cutis includes calcified subepidermal nodule, scrotal calcinosis, and tumoral calcinosis [1]. Furthermore, calcinosis cutis is classified as calcinosis circumscripta and calcinosis universalis. Calcinosis circumscripta is limited to a joint or an extremity, while calcinosis universalis has diffuse involvement of subcutaneous tissue, muscles, and tendons [1].

One of the unique aspects of our case of idiopathic calcinosis cutis was the pediatric age of the patient. Only a few cases with these characteristics have been reported in the literature. For example, Alsaif and Abduljabbarb [4] reported a case of calcinosis cutis in a seven-year-old girl with multiple nodules on the right side of her body. Gupta et al. [2] described a case of idiopathic calcinosis cutis in a 12-year-old girl, with a nodule on the right elbow. Vasudevan and Sondhi described a case of idiopathic calcinosis cutis associated with porokeratotic eccrine ostial and dermal duct nevus in an 11-year-old boy, with multiple skin lesions on the right side [3].

Extensive laboratory investigation is essential to rule out any metabolic or systemic rheumatological disorders. Investigations should include calcium and phosphorus levels, renal function tests, vitamin D levels, and serological markers of connective tissue diseases [3,4]. In our case, all of the aforementioned laboratory values revealed normal results. Furthermore, the patient had no history of previous trauma and did not report any constitutional symptoms, which confirms the idiopathic nature of the disease. It is crucial to identify the correct type of calcinosis cutis to select the optimal management options.

In this case, the patient underwent a complete excisional biopsy of the lesion and did not have any recurrence. The management of idiopathic calcinosis cutis can be difficult in pediatric patients because the medical treatments of calcinosis cutis might be associated with adverse effects that outweigh their benefits. The medical treatments used in adults include warfarin, probenecid, and colchicine [4]. Intralesional corticosteroid injection is a safe and beneficial option in children [4]. Bisphosphonates can be an effective treatment, and they act by decreasing calcium turnover [5]. Aluminum hydroxide works as a phosphate binder and might be a beneficial option in patients with hyperphosphatemia [4]. However, it should be noted that the recurrence of this disease is not uncommon [1-3].

## Conclusions

Idiopathic calcinosis cutis is a rare condition in children, particularly if it has a unilateral distribution. Proper evaluation by careful history taking, comprehensive physical examination, and laboratory workup should be performed to rule out any associated metabolic or systemic disorders that may alter the management pathway. The case highlighted the successful treatment of calcinosis cutis with excision of the lesion without the use of medical treatment, and the patient did not have a recurrence after two years of follow-up.
